# Uniform blue emitting carbon nanodots synthesized from fig fruit using reverse diffusion purification

**DOI:** 10.1038/s41598-024-80452-9

**Published:** 2024-11-26

**Authors:** Jamaan E. Alassafi, Yas Al-Hadeethi, Mohammed S. Aida, Iman S. Roqan, Samar F. Al-Shehri, Mohammad S. Ansari, Samer Alamodi, Mingguang Chen

**Affiliations:** 1 Physics Department, Faculty of Science, King Abdulaziz University, Jeddah, 21589 Saudi Arabia; 2 Center of Nanotechnology, King Abdulaziz University, Jeddah, Saudi Arabia; 3 Biochemistry department, King Abdulaziz University, Jeddah, Saudi Arabia; 4 Physical Science and Engineering Division, King Abdullah University of Science and Technology (KAUST), Thuwal, 23955-6900 Saudi Arabia; 5 Department of Chemical and Environmental Engineering, University of California, Riverside, Riverside, 92521 CA USA; 6 Asser, Health Cluster, Ministry of Health, Abha, Saudi Arabia; 7 Department of Physics, College of Science, University of Bisha, P.O. Box 344, Bisha, Saudi Arabia

**Keywords:** Blue emission, Narrow bandwidth, Green carbon sources, Pyrolysis synthesis, Reverse diffusion technique, Nanoscale devices, Nanoscale materials, Other nanotechnology

## Abstract

In this investigation, blue-emitting carbon nanodots (B.CNDs) with exceptional color purity were successfully synthesized from fresh fig fruit using a one-step pyrolysis method. These B.CNDs are small and spherical (3.7 nm) with an amorphous carbon core encapsulated inside a passivated layer primarily composed of oxygen-related functional groups. They demonstrated an emission property that is independent of excitation, showing the highest emission intensity in the deep blue region at 450 nm with a narrow full-width at half maximum (FWHM) at 44 nm and a quantum yield of 15.5%. This exceptional value of FWHM is attributed to the remarkable uniformity in both morphological and chemical composition that was achieved through the utilization of the reverse diffusion technique combined with the dialysis method for purification and separation of B.CNDs. This work not only contributes to the expanding field of carbon nanomaterials but also introduces a novel and sustainable approach to fabricate CDs with unparalleled color purity and optical performance.

**Supplementary Information:**

The online version contains supplementary material available at 10.1038/s41598-024-80452-9.

## Introduction

Fluorescent carbon dots (CDs) are small spherical quantum dots less than 10 nm in size and primarily consisting of carbon with other elements on the surface^1^. The classification of CDs is based on their core structure, which includes carbon nanodots (CNDs), carbon quantum dots (CQDs), and graphene quantum dots (GQDs). Notably, CNDs are characterized by an amorphous core structure in contrast to the crystalline core structures exhibited by both GQDs and CQDs^2^. The significant attention given to CDs is due to their low cost, excellent optical properties, resistant to photobleaching and easy to functionalize, making them ideal for cutting-edge technologies such as supercapacitors and bio-imaging^3–5^.

Since their discovery in 2004^1^, numerous fluorescent CDs covering a wide range of the visible spectrum have been synthesized from natural and chemical sources using various manufacturing methods^4–8^. Recently, extensive research efforts have been directed towards transforming green carbon sources into a value-added product of high-quality fluorescent CDs^9^, which involves applying the bottom-up synthesis methods using simple techniques such as the hydrothermal, microwave, pyrolysis, and ultrasonic^4,10,11^. For example, CDs used for biological imaging have been extracted from garlic using the hydrothermal technique^12^, papaya seeds for ion detection through the microwave method^13^, lychee seeds for cellular imaging by pyrolysis^14^ and so forth^7,8^. Even though using renewable green carbon sources and simple synthesis methods offer advantages, many CDs showed a FWHM greater than 100 nm due to diverse morphologies and chemical compositions, which limits their application in advanced optics^15^.

Currently, limited reports available on CDs produced from various carbon sources exhibiting superior color purity in blue light emission with a narrow FWHM less than 60 nm, as shown in Table 1^16^. For example, blue-emissive CDs with a narrow FWHM of 55 nm have been produced from 2,4-diaminotoluene for biolabeling^17^, and multicolour triangular CDs with emission spanning from blue to red with high colour purity (FWHM 29–30 nm) produced for light-emitting diodes derived from PG triangulogen^18^. In addition, deep-blue light-emitting materials with high color purity are produced through the utilization of amine-based edge passivation, resulting in a narrow FWHM of 35 nm and a color coordinate that closely matches the standard color specification^19^.

**Table 1:** Recent research on blue emissive CDs demonstrates the narrowest emission bandwidths.

#	Precursor	Synthesis method	Emission (nm)	FWHM (nm)	Reference
**1**	Phloroglucinol	Solvothermal	472	30	[^18^]
**2**	Phloroglucinol	Glycol Solvent	481	30	[^20^]
**3**	Citric acid and diaminonaphthalene	Solvothermal	433	35	[^19^]
**4**	2,4-Diaminotoluene	Ambient Temperature	490	55	[^21^]
**5**	Glycols and H_2_SO_4_	Solvothermal	400	59	[^22^]
**6**	Fresh fig fruit	Pyrolysis	450	44	This work

To date, most research on CDs have primarily focused on synthesis, characterizations and applications with minimal attention given to purification and separation processes. Nevertheless, CDs synthesis generally results in a combination of diverse CDs fractions of varying sizes, alongside ultra-small luminescent fluorophores and non-fluorescent nanoparticle by-products. Therefore, it is crucial to carry out purification and separation techniques on the prepared CDs solution to regulate their photoluminescence (PL) emission and enhance their emission purity. One common approach is using dialysis to purify the CD solution from small fluorescent molecules by employing a membrane with a specific cut-off size, while size separation is achieved by selecting a dialysis bag with a suitable molecular weight cut-off membrane^23,24^.

In this study, a novel methodology was used to fabricate renewable, cost-efficient, and high-purity fluorescent B.CNDs derived from fig fruits through a pyrolysis technique. The method of reverse diffusion combined with a suitable dialysis bag was employed to effectively isolate and collect B.CNDs that demonstrate uniform optical properties.

The technique of reverse diffusion was utilized to facilitate the movement of nanoparticles towards an area of lower concentration within the dialysis bag, employing two different dialysis membranes for the purification and collection of B.CNDs from the synthesized solution. Initially, a small-scale dialysis bag with a molecular weight cutoff of 0.5 k Da was implemented to effectively remove ultra-small fluorophores from the solution. Following this, a larger dialysis bag with a molecular weight cutoff of 1.0 k Da was employed to collect B.CNDs, which displayed remarkable uniformity with respect to their morphological and chemical characteristics. This consistent uniformity led to the generation of B.CNDs with homogeneous optical properties, as evidenced by the narrow emission bandwidth associated with their excitation-independent emission properties.

## Experimental methods

### Materials

Local fresh fig fruits obtained from the local market and high-purity water were used in the experiment.

### Synthesis of the B.CNDs

**Fig. 1:**
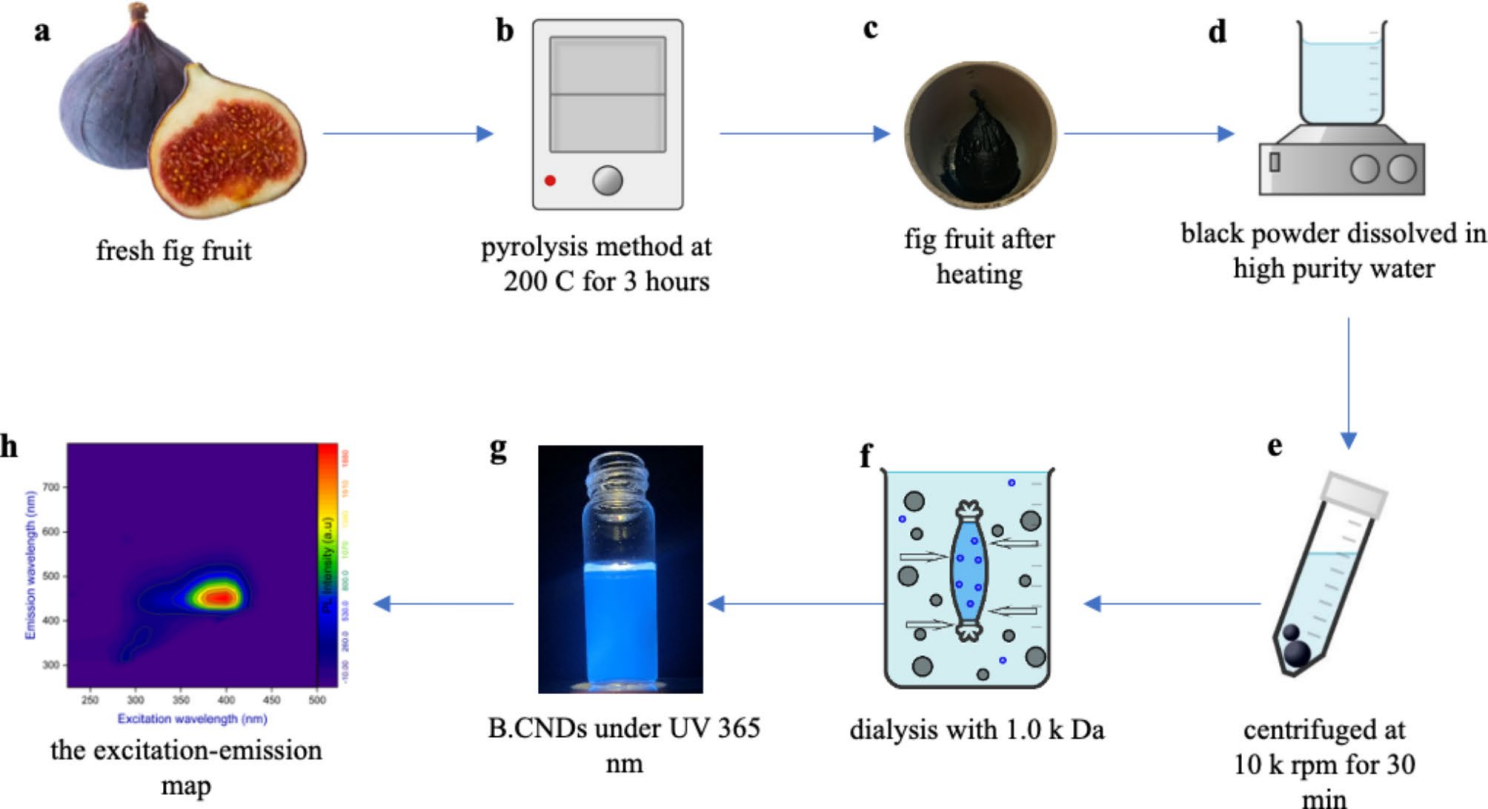
The schematic diagram of the experimental procedure to synthesize B.CNDs, as confirmed by the excitation-emission map of the diluted B.CNDs in water (Fig. 2.h).

The fig fruit is nutritious, rich in carbohydrates, sugars, and organic acids that can be used as carbon sources^25^. In this study, local fresh figs were used to obtain CDs through the pyrolysis method as shown in Fig. 1.

First, 5 g of fig fruits were subjected to a cleansing process using distilled water to eliminate any remnants of dust and dirt, followed by a thermal treatment in an oven set at a temperature of 200 ºC for a duration of three hours. After cooling to room temperature, a black powder was extracted solely from the inner part of the fig fruit (Fig. 1.c) without including the components of the outer shell to minimize environmental contaminants during the preparation and carbonation processes. The black powder was then crushed using a blender and dissolved in ultrapure water for one hour with the aid of an ultrasonic bath. The supernatant was subjected to filtration through a 0.22 μm syringe filter followed by centrifugation at 10 k rounds per minute (rpm) for half an hour to eliminate large undissolved particles. The yellowish-solution underwent dialysis for purification. The purified product turned dark blue under UV light at 365 nm (as shown in Fig. 1.g).

**Fig. 2:**
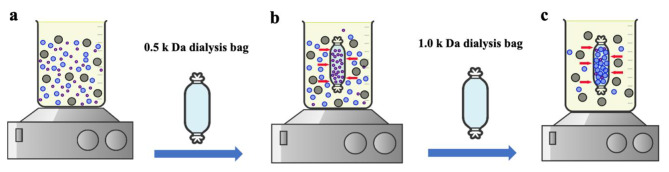
The schematic illustration of the purification and separation process of the B.CND solution. (**a**) CD solution after being filtered by 0.22 μm and centrifuged at 10 k rpm for 30 min. (**b**) CDs purified with a 0.5 k Da dialysis bag. (**c**) B.CNDs extracted using a 1.0 k Da dialysis bag.

Here, the uniformity in the optical properties of B.CNDs was achieved using low molecular weight cutoff (0.5 k Da and 1.0 k Da) dialysis membranes, Fig. 2. Initially, the yellowish-solution of CDs was placed in a 100-mL glass tube, followed by the insertion of a dialysis bag (0.5 k Da) containing ultrapure water into the tube. This technique facilitated the reverse diffusion of ultra-small nanoparticles into the ultrapure water inside the dialysis bag, successfully isolating them from the yellowish-solution of CDs. The ultrapure water within the dialysis bag underwent continuous replacement every 8 h until no emission was visible from the water expelled under UV light at 365 nm, indicating the effective elimination of nearly all ultra-small nanoparticles. The same procedure was repeated but with using a dialysis bag of higher molecular weight (1.0 k Da) to aid in the diffusion of B.CNDs into the dialysis bag, while retaining larger and non-fluorescent nanoparticles in the synthesized solution. The water containing B.CNDs in a dialysis bag was freeze-dried under vacuum to produce B.CND powder that was stored at ambient conditions for additional analysis.

### Characterization of the B.CNDs

The solution was purified using two types of dialysis kits. First, it was subjected to a dialysis kit with a molecular weight cut-off of 0.5 k Da (Float-A-Lyzer G235061 Dialysis, Japan). Then, a cellulose dialysis bag tubing with a MWCO of 1.0 k Da was used for further purification (Linyeue, China).

A scanning electron microscope (SEM) and a high-resolution transmission electron microscopy (HR-TEM) were utilized to examine the morphology and size of B.CNDs. The process involved adding B.CNDs solution to ultrapure water, sonicating it, placing a drop on a silicon substrate, and analyzing it with SEM model JSM-7600 F (JEOL, Tokyo, Japan). The same procedure was followed for TEM measurements, using TEM model Jem-2100f (Jeol, Tokyo, Japan) operated at 200 kV and a thin carbon-coated copper grid.

The examination of the crystallinity structure was conducted through the utilization of X-ray diffraction analysis (XRD) using an Ultima-IV system equipped with a Cu X-ray tube operating at 40 kV and 40 mA with scanning speed at 4° min-1 and covering a 10°– 80° range.

The surface charge and size distribution of B.CNDs were studied by Zeta-sizer (nano-ZS Zetasizer, Malvern Instruments Ltd). A single-use cuvette specific to the instrument was used for sample preparation and the measurement was conducted at ambient temperature with a pH value of 7.

The surface composition of B.CNDs was examined using X-ray photoelectron spectroscopy (XPS) model K-Alpha Plus (Thermo, USA) with CasaXPS software. Fourier transform infrared (FTIR) was also utilized for surface composition examination. The instruments used were model K-Alpha Plus and Nicolet iS FTIR spectrometer (Thermo, USA).

UV-visible spectroscopy (UV-3600; Shimadzu, Japan) and fluorescence spectrophotometry (F-7000; Hitachi, Japan) were used to analyze B.CNDs absorption spectra and PL measurements, respectively, with all measurements conducted in a quartz cuvette with a 10 mm pathlength at ambient temperature.

The photostability of B.CNDs was analyzed under UV light for a period of 60 min employing a Camag UV Cabinet II that had a 55 W UV source emitting light at a wavelength of 365 nm.

The quantum yield (QY) of the B.CNDs was calculated using a single point of relative method^26^. The decision to select quinine sulfate as a reference with a known QY of 0.54 in 0.1 M H_2_SO_4_ was based on its similar absorption and emission wavelengths to B.CNDs, along with keeping absorbance concentrations below 0.1 to minimize reabsorption. After that, the PL spectra and integrated emission intensities were measured under excitation at a wavelength of 350 nm. The QY of B.CNDs in pure water was determined to be 15.5% and calculated using a following Eq. (1):

1 $$\Phi = {\Phi _R} \times \:\frac{{{{\text{G}}_S}}}{{{{\text{G}}_R}}} \times \:\frac{{{{\text{A}}_S}}}{{{{\text{A}}_R}}} \times \:\frac{{\eta _S^2}}{{{\eta _R}^2}}$$

The symbols Φ, G, and A are used to indicate QY, integrated fluorescence intensity, and absorbance, respectively. The symbol η is employed for the representation of the refractive index, which is set at a value of 1.33 for both H_2_SO_4_ and water. The symbol R is employed to indicate the reference standard possessing a known QY, while terms with the symbol S are associated with the synthesized B.CNDs.

## Results and discussion

In the literature, it has been reported that the majority of reported CDs from green sources were obtained by chance rather than design due to limitations in controlling the final properties of CDs through existing synthesis methods^27^. The complexity of the structure and chemical composition of the green precursors may be the contributing factor to the variability in physical and electronic properties that are observed in the resulting CDs. In the present investigation, attempts were undertaken to isolate and refine a set of B.CNDs possessing homogeneous optical characteristics. Subsequently, these B.CNDs underwent comprehensive analysis methodologies to investigate in depth their size distribution, core configuration, chemical composition and optical properties.

### Morphology and chemical composition of B.CNDs

**Fig. 3:**
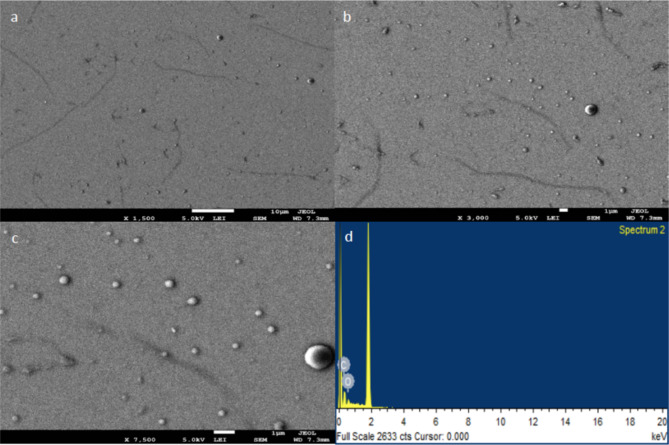
(**a**-**c**) SEM images show well-distributed spherical carbon nanoparticles. (**d**) the EDS spectrum shows only carbon and oxygen element on silicon substrate.

**Fig. 4:**
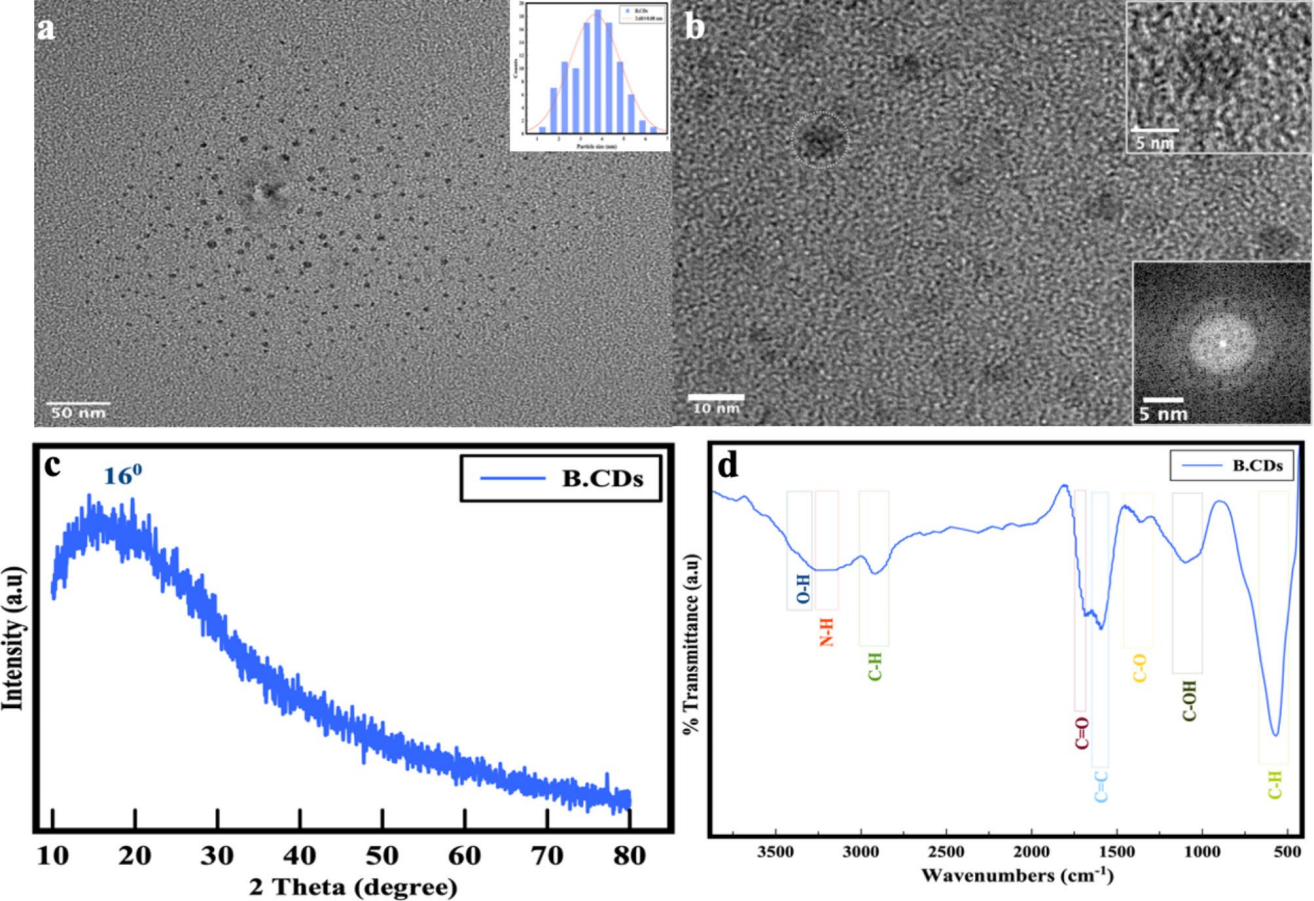
Structural and chemical composition properties of B.CNDs. (**a**) TEM image of the B.CNDs shows a wide range of particle distribution (top inset image). (**b**) a low contrast TEM image between B.CNDs and a thin carbon-coated copper of TEM grid shows a poor contrast between B.CNDs and TEM grid (upper inset image) and FFT pattern demonstrating their amorphous structure (lower inset image). (**c**) XRD of B.CNDs shows its amorphous. (**d**) FT-IR of B.CNDs shows their surface are rich with oxygen and some nitrogen related function groups.

The morphology and size distribution of B.CNDs were examined using SEM and TEM, as shown in Figs. 3 and 4. Data from both SEM and TEM indicate the presence of nanoparticles with spherical-like shape and consistent size distribution over a large area.

Figure 3.a-c showed well-distributed spherical nanoparticles with elemental composition analyzed by energy dispersive X-ray spectroscopy (EDS) (Fig. 3.d). Results indicated carbon as the main component, with some oxygen, confirming no other foreign elements were presented on the TEM grid. Nevertheless, SEM visualized B.CNDs and surface composition, but not structural details of the carbon core.

To overcome this limitation, a TEM microscopic approach is used to estimate size distribution and investigate the internal structural characteristics of B.CNDs. Formation of black clusters of B.CNDs in sizes ranging between 1 and 6.5 nm with an average diameter of 3.7± 0.1 is shown in Fig. 4.a, and observing clear images of B.CNDs is challenging due to poor contrast with TEM grids coated with thin amorphous carbon. In addition, the upper inset image of HR-TEM image in Fig. 4.b shows a dark spot of B.CNDs without clear lattice fringes. The Fast Fourier Transform (FFT) pattern in the lower inset also indicates a spherical morphology with no defined diffraction fringes, confirming the amorphous nature of B.CNDs.

The disorder-like structure of B.CNDs indicated by HR-TEM is confirmed through XRD analysis since it solely provides information about the core structure, as illustrated in Fig. 4.c. XRD pattern indicates a broad peak at 2θ = 16° corresponding to an interlayer spacing of 0.55 nm, suggesting the amorphous nature of B.CNDs^28–31^. The broad peak pattern may result from small sizes and oxygen-containing functionalities on surfaces, leading to broadening and shifting of the graphitic signal structure to a lower angle around 16°^28,32^. Therefore, both HR-TEM and XRD techniques confirm that the core of the B.CNDs is amorphous and lacks any graphitic like-structure.

**Fig. 5:**
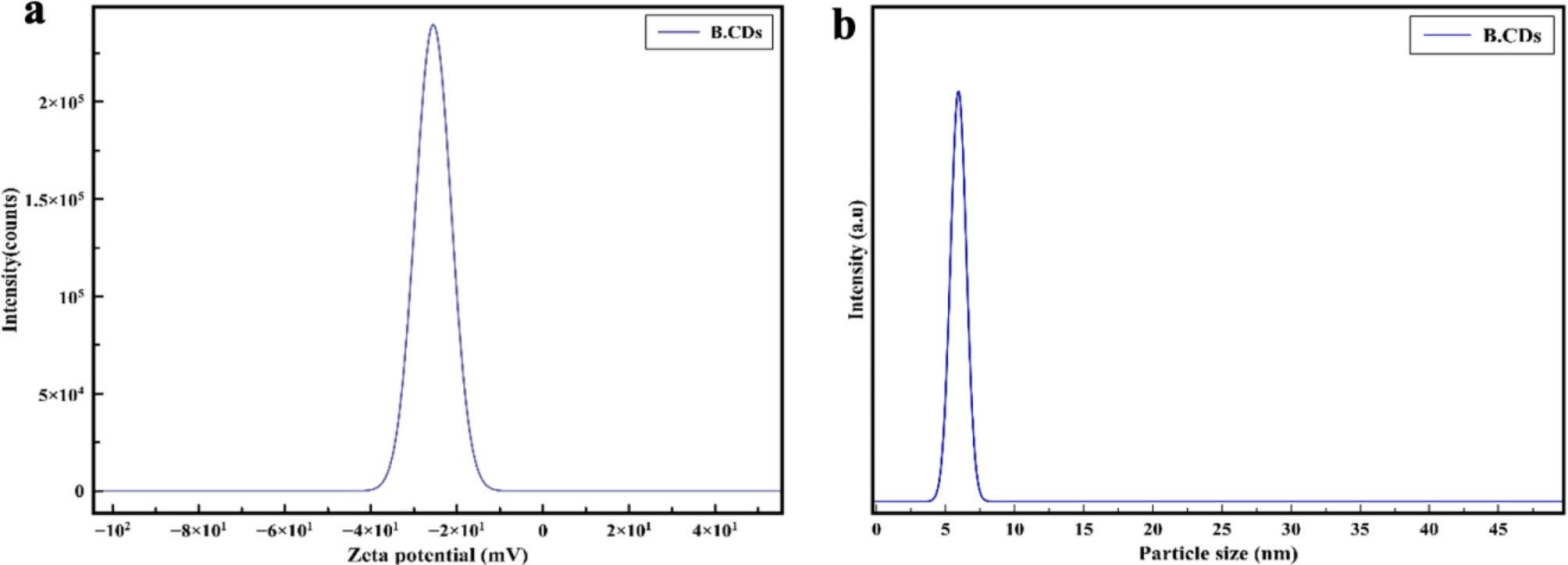
(**a**) Dynamic light scattering (DLS) size distribution curve of B.CNDs dissolved in water. (**b**) the zeta potential curve of B.CNDs in water shows a negative charge surface.

To confirm the size distribution of B.CNDs, dynamic light scattering (DLS) is used due to its ability to analyze a higher number of particles compared to TEM. The average size distribution of B.CNDs was found to be slightly larger (5.8 ± 0.5 nm) than that obtained by TEM (3.7± 0.1 nm), Fig. 5.a. In fact, the hydrodynamic diameter of CDs in solution is always greater than the diameter determined in a dry form under high vacuum conditions by TEM, which could be attributed to the presence of multi-functional groups on the surface of B.CNDs^33^.

The presence of these multifunctional groups on the surface of B.CNDs was further confirmed by the zeta potential method that shows a negative potential peak at -28 mV (Fig. 5.b) aiding efficient dispersion in water-based solvents and stability. Nonetheless, zeta potential analysis can provide details on the overall surface charge but does not indicate the specific functional groups present on the surface of B.CNDs.

Consequently, FT-IR spectroscopy technique was utilized to examine the chemical compositions and functional groups on the surface of B.CNDs, as depicted in Fig. 4.d. The FT-IR spectrum indicates various functional groups related to oxygen and nitrogen on the surface of B.CNDs through absorption peaks at specific wavenumbers at 3216, 2925, 1690, 1592, 1331, 1030 and 620 cm^− 1^.

A peak of absorption at 3216 cm^− 1^ was detected indicating the existence of O-H and N-H bonds, thereby verifying the existence of oxygen and nitrogen-associated functional moieties on the B.CNDs surface^34^. A stretching vibration at 2925 cm^− 1^ confirms the presence of CH_2_ symmetry and suggests that the hydrocarbon in the fig fruit precursor has not been fully carbonized, and this can be accomplished by providing more thermal energy^35^. Similarly, the stretching peaks at 1701 cm^− 1^ show the C = O vibration of the carboxylic group, while peaks at 1602 cm^− 1^ show C = C vibration demonstrate the establishment and reinforcement of the carbon core structure^34,36,37^. Furthermore, peaks observed at 1331 and 1030 cm^− 1^ are indicative of the presence of C-O and C-OH groups, respectively^34,37,38^. Meanwhile, the peak detected at 620 cm^− 1^ can be attributed to the aromatic C-H bending in benzene^39^.

Overall, the FT-IR spectra displayed a carbonized core on B.CNDs with polar functional groups such as hydroxyl, amine, and carboxyl, improving water dispersibility and validating prior DLS results. Nonetheless, the FT-IR lacks the capability to provide more detailed information about the quantity of each individual functional group on the surface of B.CNDs.

**Fig. 6:**
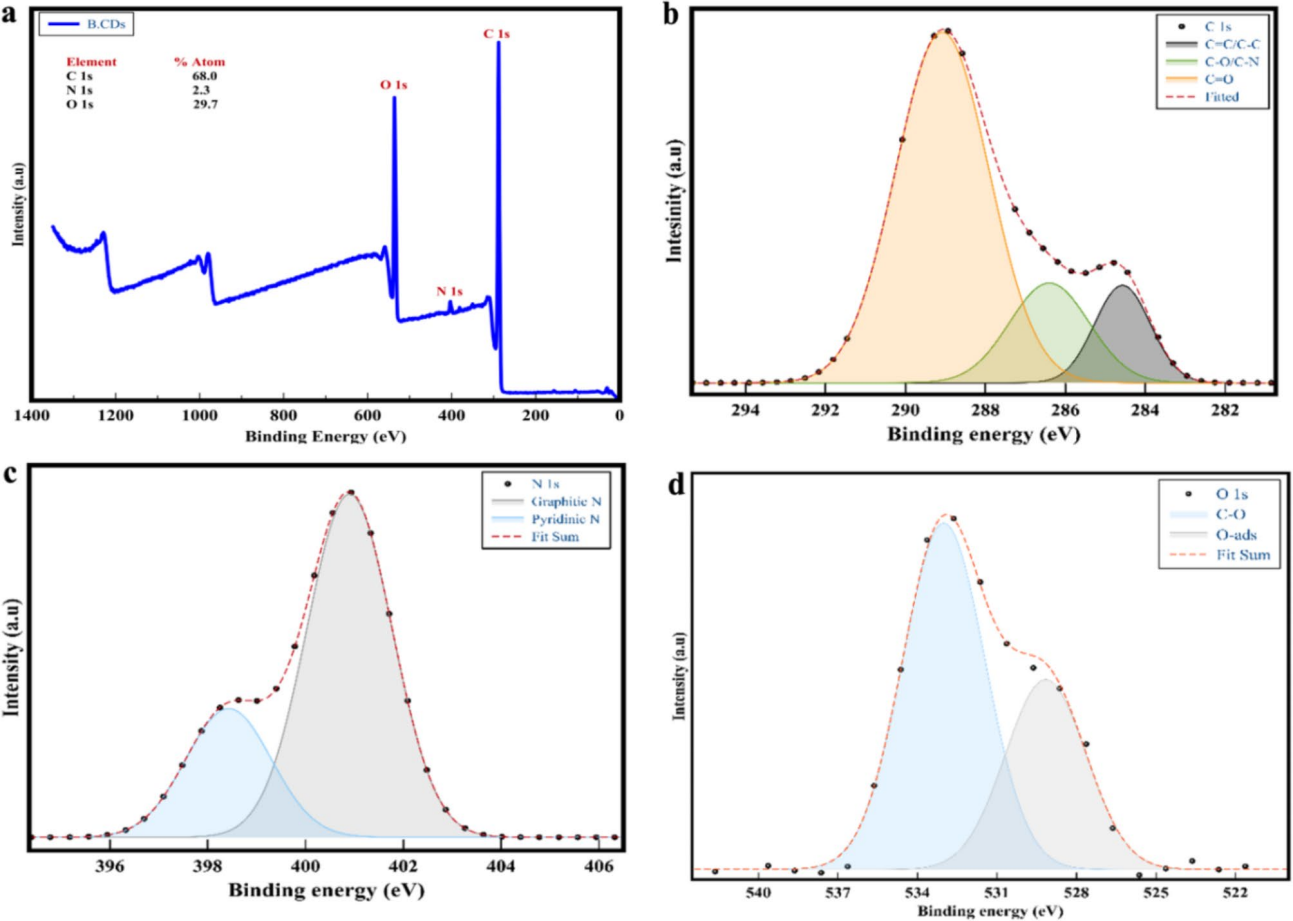
(**a**) XPS spectra of as-synthesized B.CNDs shows it consists mainly of carbon, nitrogen and oxygen. (**b**) High-resolution XPS of C 1s. (**c**) High-resolution XPS of N 1s. (**d**) High-resolution XPS of O 1s.

Therefore, the application of the quantitative technique of the XPS was utilized for the purpose of determining a precise composition of oxygen and nitrogen functional groups within a depth of 10 nm from the surface of B.CNDs, as demonstrated in Fig. 6. The XPS analysis reveals that the surface layer of the B.CNDs is primarily composed of carbon (68.0%), nitrogen (2.3%), and oxygen (29.7%) as illustrated in Fig. 6.a. No additional components were identified, indicating the high quality of the carbon source that was utilized. Nevertheless, the EDS analysis solely identified carbon and oxygen, in contrast to the information obtained from XPS. This discrepancy could be attributed to the higher sensitivity of XPS towards lighter elements such as nitrogen, which exists in a very minimal concentration. Moreover, the data derived from a limited number of nanoparticles in EDS may not accurately represent the entire sample as demonstrated in XPS.

The high-resolution XPS of C 1s (Fig. 6.b) sheds light on the formation of diverse carbon bond configurations. The C 1s spectrum in HR-XPS can be resolved into three Gaussian peaks located at 284.2 eV (22.3%), representing the (sp^2^) bond in graphite structure C = C, a smaller peak at 286.6 eV (20.1%) attributed to the (sp^3^) bond of C-O/C-N, and the primary peak at 288.6 eV (57.6%) corresponding to the (sp^2^) bond of C = O and O-C = O^40^. The HR- XPS of N 1s (Fig. 6.c) reveal the existence of two peaks at 398.5 eV (26.2%) that could be assigned to Pyridinic N functional group and another major peak at 401.4 eV (73.8%) indicates that nitrogen was introduced into the structure of the graphite to create graphitic nitrogen doping^41^. Additionally, HR-XPS of O 1s (Fig. 6.d) is resolved into two peaks: the dominant peak at 533.1 eV (66.0%) representing C-O and a smaller peak at 529.3 eV (34.0%) corresponding to O-ads^42^. Based on the XPS analysis conducted, it can be deduced that the B.CNDs surface encompasses various oxygen and nitrogen-related functional groups such as hydroxyl, carboxylic, and amino groups. This finding corroborates the outcomes obtained from the FT-IR spectra analysis.

By combining all these characterization data, the B.CNDs consists of a spherical carbon nanoscale with an amorphous core enclosed in an oxygen-based shell, primarily (C = O).

### Optical properties of the B.CNDs

**Fig. 7:**
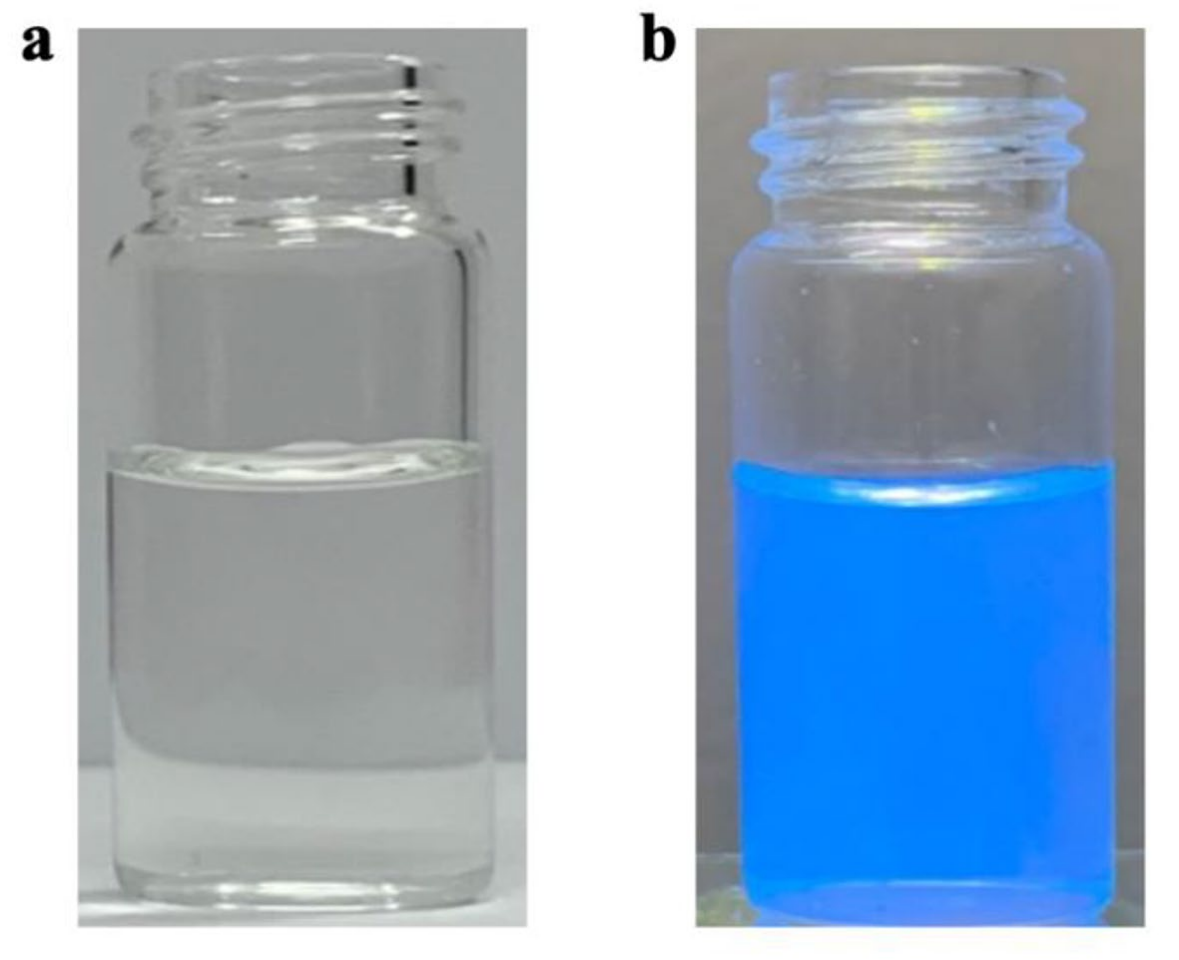
Photograph images of the B.CNDs depressed in water (**a**) normal daylight and (**b**) normal daylight under UV light at 365 nm.

The optical properties of the B.CNDs were analyzed in high-purity water at room temperature. The B.CNDs appear transparent in daylight and turn dark blue under UV light at 365 nm as illustrated in Fig. 7.

**Fig. 8:**
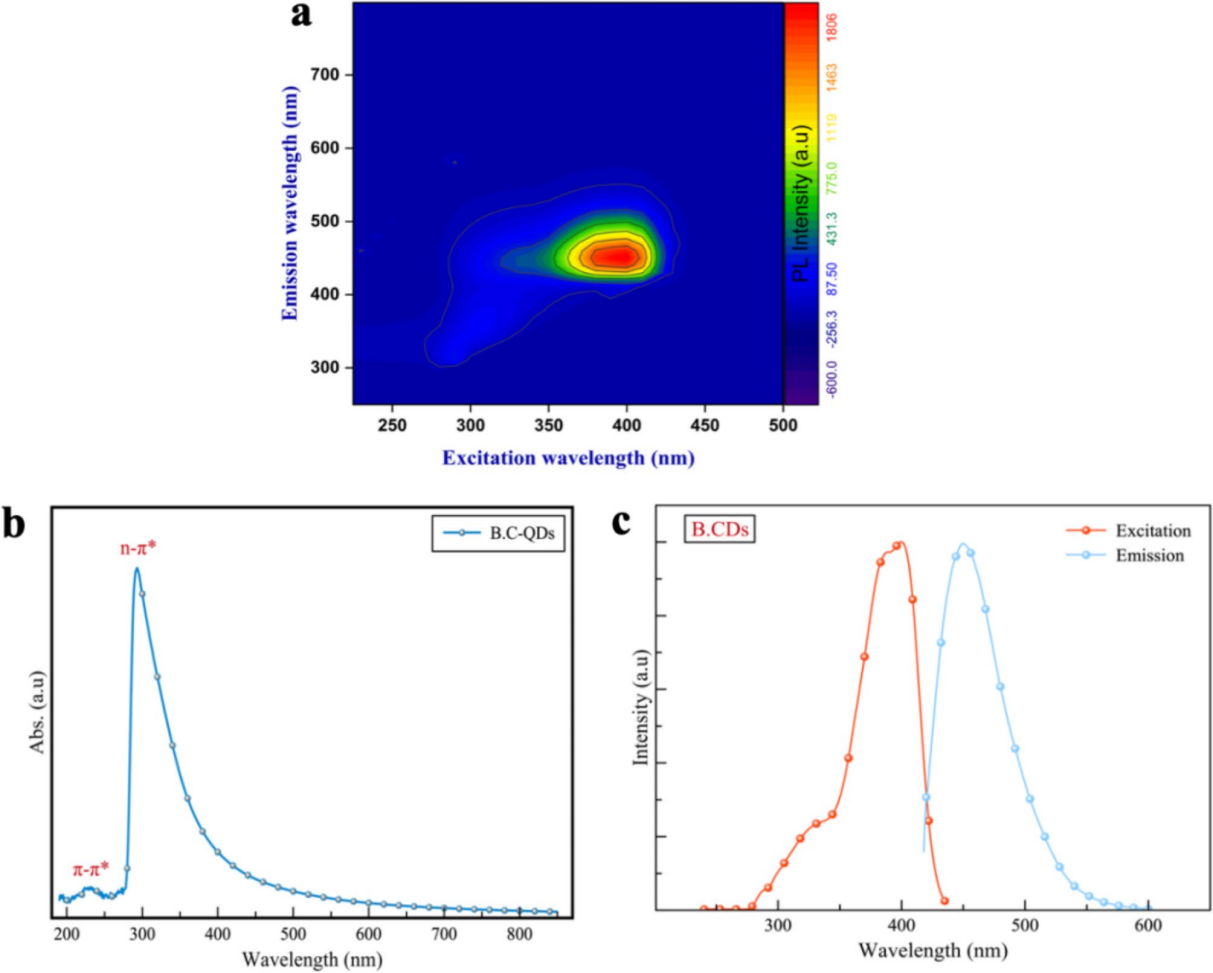
Optical properties of the B.CNDs in in high purity water (**a**) Excitation-emission color map (**b**) Optical absorption spectrum, (2) normalized spectrum of PL excitation (400 nm), and PL emission (450 nm).

The excitation-emission map of the B.CNDs is shown in Fig. 8.a. A singular emission wavelength of 450 nm is detected when using excitation wavelengths ranging from 300 to 420 nm and no measurable fluorescence is observed with excitation wavelengths below 270 nm or above 420 nm due to the absence of active emission centers in those regions.

The UV-Vis spectrum of B.CNDs (Fig. 8.b) exhibits optical absorption primarily in the ultraviolet (UV) region, with some absorption continuing into the visible region. The UV-Vis spectroscopy revealed the presence of two absorption peaks at 225 nm (5.5 eV) induces the π-π* transition of sp^2^ hybridized from the carbon core (C = C) embedded in the dominated sp^3^ matrix located at 288 nm (4.3 eV) that could be related to n-π* transition of surface groups, probably (C = O)^43,44^. This is consistent with FTIR and XPS data showing the presence and dominance of C = O on the surface of B.CNDs.

Absorption measurements provide details on ground to excited state transitions, while photoluminescence excitation (PLE) reveals energy levels associated with particular light emission bands. The PLE spectrum (Fig. 8.c) displays two excitation peaks at 450 nm emission wavelength. A minor peak is observed at 347 nm (3.6 eV) and a major sharp peak at 393 nm (3.1 eV). The first peak can be linked to the carbon core absorption band, while the latter can be associated with C = O surface groups. Overall, the PLE curve suggests a 450 nm emission peak primarily from a 3.1 eV transition, with a small contribution from a 3.6 eV transition.

The PL spectrum (Fig. 8.c) illustrates the emission properties of the B.CNDs in the wavelength range between 418 and 575 nm, with a maximum emission intensity at 450 nm, a QY of 15.5% and a narrow FWHM of 44 nm, surpassing previously reported values for conventional CDs. The exceptional values of FWHM outperform those reported for CDs derived from green sources, a phenomenon that can be ascribed to the remarkable uniformity in both morphology and chemical composition of the B.CNDs^45,46^.

Overall, the absorption curve and PLE spectra align and complement each other, suggesting that the emission of B.CNDs at 450 nm originates from two electronic transitions due to carbon core and surface defects.

### PL mechanism

The PL mechanism of CDs remains a topic of debate with no universal mechanism established^47,48^. Nevertheless, The two widely accepted PL mechanisms include excitation-independent and excitation-dependent mechanisms^49^. The excitation-independent emission is due to a single electronic transition when the excitation wavelength changes, caused by fluorophores and fully passivated CDs^49,50^. On the contrary, the occurrence of excitation-dependent emission has been attributed to quantum confinement, heteroatom electronegativity, and surface trapping. This phenomenon is characterized by a displacement of emission peaks towards longer wavelengths upon excitation with longer wavelengths^43,48,51–53^. Quantum confinement effects are particularly significant in nanoscale materials like CDs, where electrons are confined in a small space. This confinement leads to quantization of energy levels, resulting in size and shape-dependent optical properties. Heteroatom electronegativity and surface trapping also play crucial roles by introducing localized energy states and trapping sites, which can alter the emission properties based on the excitation conditions^51–53^.

**Fig. 9:**
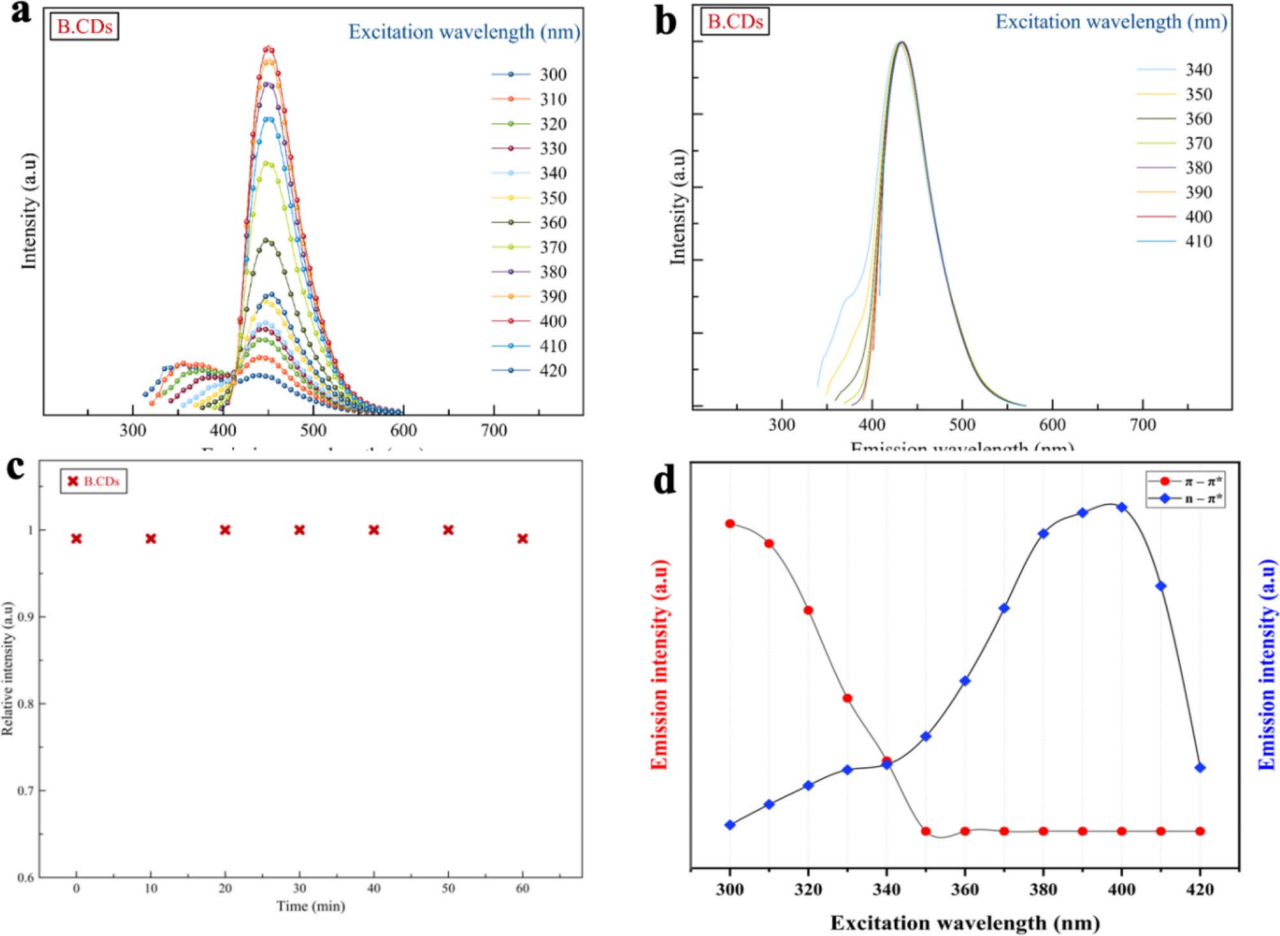
(**a**) Photoluminescence spectrum of the B.CNDs with increasing of excitation wavelengths from 300 to 420 nm (**b**) normalized PL spectra of the B.CNDs. (**c**) Photostability of the B.CNDs after continued irradiation with a 55 W xenon lamp for 60 min at wavelength of 365 nm. (**d**) Excitation-emission relation for both UV and blue region of B.CNDs.

Figure 9a demonstrates a symmetric PL spectra shape of B.CNDs exhibiting blue emission with excitation-independent property, aligning with existing literature^29,40,48^. The peak position of the PL spectra remains unchanged at 450 nm upon changing the excitation wavelength between 340 and 420 nm, (Fig. 9.b). Moreover, the intensity initially increases as the excitation wavelengths increased until peaking at 400 nm, followed by a gradual decrease at longer wavelengths. Nevertheless, the PL of B.CNDs (Fig. 9.c) remains stable even after exposure to a 55 W xenon lamp for 60 min at 365 nm, indicating high photostability. This stability may be attributed to the high degree of carbonization shown in EDS and XPS data^54^.

Figure 9.d clearly illustrate the presence of two emissive centers that contribute to the emission spectra of B.CNDs when excited within the wavelength range of 300 to 420 nm. This observation aligns well with the data obtained from absorption and PLE spectra. The small first emission center (π-π*) displays excitation-dependent behavior associated with the carbon core due to quantum-confinement, leading to a red-shift in emission and decreasing intensities as excitation wavelengths change from 300 to 350 nm. Conversely, the dominant emission center (n-π*) linked to surface states became predominant beyond the excitation wavelength of 350 nm, with a steady increase in their intensities demonstrating an excitation-independent property.

As stated earlier, the peak positions of the B.CNDs remain constant at 450 nm, differing from highly crystalline CDs that exhibit excitation-dependent emission and display red-shifted peak positions with changing excitation wavelength toward longer regions^48^. This constancy can be explained by the quantum mechanical phenomenon of energy level stabilization in amorphous structures. In crystalline structures, the regular atomic arrangement at nanoscale leads to a clear set of quantized energy levels, resulting in excitation-dependent emission. In contrast, the amorphous nature of the B.CNDs leads to a more randomized energy level distribution, which can stabilize the emission wavelength and reduce the dependence on the excitation wavelength^55–57^.

Analytical techniques reveal B.CNDs have an amorphous core and shell with oxygen and nitrogen-functional groups. The shell is passivated uniformly with oxygen and some nitrogen-functional groups have electron-withdrawing (C = O, N = O) and electron-donating functional groups (-OH, -NH)^58,59^. Moreover, the presence of oxygen and nitrogen functional groups in B.CNDs introduces discrete energy levels within the bandgap, causing extra recombination pathways apart from band-to-band recombination^59–61,61^. Nevertheless, based on XPS data analysis, our sample has fewer N-related functional groups than O-related functional groups, suggesting nitrogen has a smaller impact on PL compared to oxygen-related functional groups.

Combining all the characterization data, the B.CNDs consist of a disordered carbon core with a uniformly passivated amorphous shell layer rich in oxygen-related functional groups, primarily (C = O). This uniform surface passivation decreases bandgap energy, resulting in a single excitation-independent emission mode with blue emission at 450 nm. The passivated shell serves as a barrier, altering energy levels of electrons and holes, leading to a narrower energy gap^62–64^. The proposed mechanism for electron and hole recombination involves excitation through the co-existence of π - π* and n - π* absorption bands and relaxation through a non-radiative transition. Finally, radiative recombination occurs through the C = O channel, resulting in a single-mode transition with strong symmetric emission peaks at 450 nm and a narrow FWHM at 44 nm.

## Conclusion

The method of pyrolysis was employed to produce blue carbon nanodots (B.CNDs) with high-color purity and a narrow emission bandwidth from newly harvested figs. Utilizing a novel reverse diffusion technique combined with a dialysis method for purification and separation generates B.CNDs with uniform optical characteristics. These B.CNDs displayed a prominent emission peak in the deep blue region at 450 nm, accompanied by a QY of 15.5% and an exceptionally narrow FWHM at 44 nm. The B.CNDs have a strong negative surface charge due to oxygen-related functional groups, which endowed them with superior water dispersibility. This study presents a novel method for producing high-quality CDs suitable for advanced optical applications. The research contributes significantly to the realm of carbon nanomaterials, especially in addressing the increasing demand for precise optical properties in CD materials. CD materials hold promise for influencing advanced optical technologies such as photoelectric devices, sensor detectors, and bio-imaging applications.

## Electronic supplementary material

Below is the link to the electronic supplementary material.

Supplementary Material 1

## Data Availability

All data generated or analyzed during this study are included in this published article [and its supplementary information files].
