# Supplementary material for: Uniform blue emitting carbon nanodots synthesized from fig fruit using reverse diffusion purification
Source: Sci Rep. 2024 Nov 26;14:29254. doi: 10.1038/s41598-024-80452-9 (PMC11589837; doi:10.1038/s41598-024-80452-9)
Supplement: Supplementary file 1 — Supplementary Material 1 [file 41598_2024_80452_MOESM1_ESM.docx]

**Supporting Information:**

**1.** Scanning electron microscopy (SEM) and energy dispersive X-ray spectroscopy (EDS) of B.CNDs


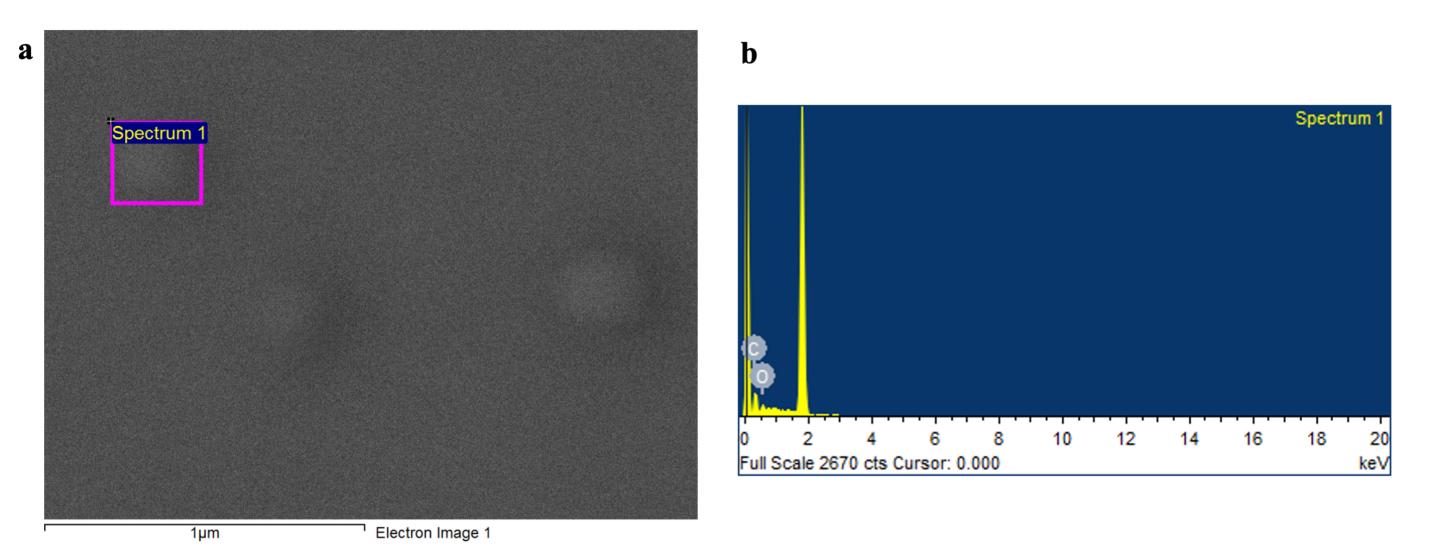


**Fig. 1 (a)** SEM images of spherical carbon nanoparticles. (d) the EDS spectrum shows only carbon (76.07%) and oxygen (23.93%) element on silicon substrate as it shown in table.

**Table 1:** EDS elemental analysis of B.CNDs shows carbon and oxygen

| **Element** | **Weight%** | **Atomic%** |
| --- | --- | --- |
| ***C K*** | ***70.47*** | ***76.07*** |
| ***O K*** | ***29.53*** | ***23.93*** |
| ***Totals*** | **100** | |

**2.** Dynamic light scattering (DLS) and Zeta potential of B.CNDs


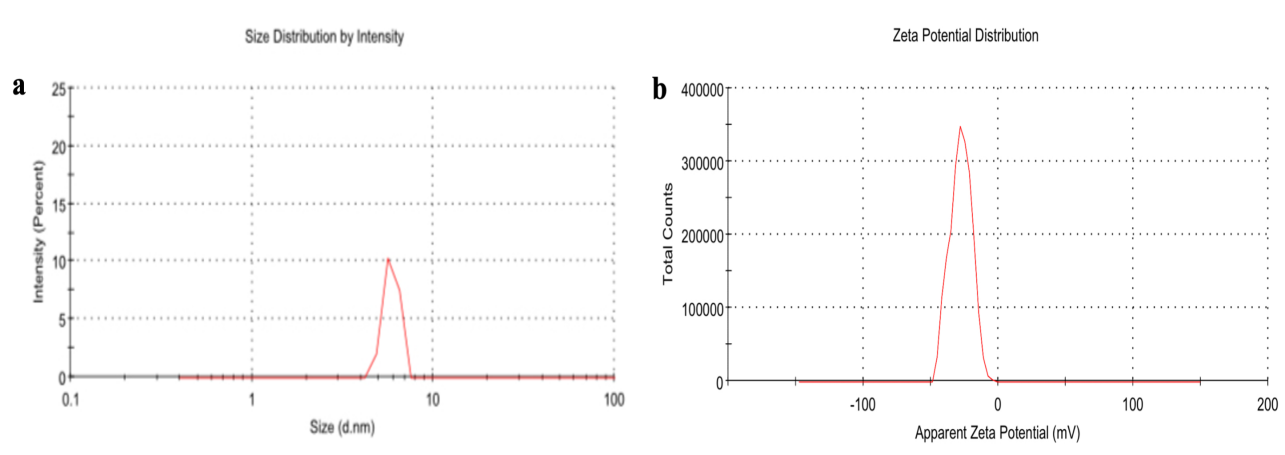


***Fig.2.*** *(a) Dynamic light scattering (DLS) size distribution curve (5.8 nm) of B.CNDs dissolved in water. (b) the zeta potential curve of B.CNDs (-28 eV) in water shows a negative charge surface.*
